# Rhein Induces Oral Cancer Cell Apoptosis and ROS via Suppresse AKT/mTOR Signaling Pathway In Vitro and In Vivo

**DOI:** 10.3390/ijms24108507

**Published:** 2023-05-09

**Authors:** Haibo Zhang, Lei Ma, Eungyung Kim, Junkoo Yi, Hai Huang, Hyeonjin Kim, Muhammad Atif Raza, Sijun Park, Soyoung Jang, Kirim Kim, Sung-Hyun Kim, Youngkyun Lee, Eunkyong Kim, Zae Young Ryoo, Myoung Ok Kim

**Affiliations:** 1 Department of Animal Science and Biotechnology, Kyungpook National University, Sangju 37224, Republic of Korea; 2 Department of Animal Biotechnology, Research Center for Horse Industry, Kyungpook National University, Sangju 37224, Republic of Korea; 3 College of Pharmacy, Henan University of Chinese Medicine, Zhengzhou 450046, China; 4 School of Animal Life Convergence Science, Hankyung National University, Anseong 17579, Republic of Korea; 5 School of Life Sciences, BK21 FOUR KNU Creative BioResearch Group, Kyungpook National University, Daegu 41566, Republic of Korea; 6 Department of Dental Hygiene, Kyungpook National University, Sangju 37224, Republic of Korea; 7 Department of Bio-Medical Analysis, Korea Polytechnic College, Chungnam 34134, Republic of Korea; 8 School of Dentistry, Kyungpook National University, Daegu 41566, Republic of Korea

**Keywords:** apoptosis, autophagy, mTOR, oral cancer, rhein

## Abstract

Oral cancer remains the leading cause of death worldwide. Rhein is a natural compound extracted from the traditional Chinese herbal medicine rhubarb, which has demonstrated therapeutic effects in various cancers. However, the specific effects of rhein on oral cancer are still unclear. This study aimed to investigate the potential anticancer activity and underlying mechanisms of rhein in oral cancer cells. The antigrowth effect of rhein in oral cancer cells was estimated by cell proliferation, soft agar colony formation, migration, and invasion assay. The cell cycle and apoptosis were detected by flow cytometry. The underlying mechanism of rhein in oral cancer cells was explored by immunoblotting. The in vivo anticancer effect was evaluated by oral cancer xenografts. Rhein significantly inhibited oral cancer cell growth by inducing apoptosis and S-phase cell cycle arrest. Rhein inhibited oral cancer cell migration and invasion through the regulation of epithelial–mesenchymal transition-related proteins. Rhein induced reactive oxygen species (ROS) accumulation in oral cancer cells to inhibit the AKT/mTOR signaling pathway. Rhein exerted anticancer activity in vitro and in vivo by inducing oral cancer cell apoptosis and ROS via the AKT/mTOR signaling pathway in oral cancer. Rhein is a potential therapeutic drug for oral cancer treatment.

## 1. Introduction

Oral cancer (OC) is a type of head and neck cancer and is one of the most common malignancies worldwide [1]. Approximately 90% of oral and oropharyngeal malignancies are squamous cell carcinomas [2]. Despite new diagnostic techniques and treatment strategies for OC treatment, the disease remains a critical challenge because of drug resistance and metastasis. Surgery is the mainstay of treatment for OC, and patients with advanced-stage cancer or who are inoperable are usually treated with combination therapy [3]. Thus, the development of more effective as well as safer drugs and novel therapies for OC treatment is an urgent task.

Autophagy is a cytoplasmic component degradation process that is paramount in cellular homeostasis and survival. Autophagy plays a dual role in tumorigenesis, although some studies have reported that autophagy exerts a pro-survival effect, most agent-induced autophagy mainly leads to cell death in cancer [4,5]. Microtubule-associated protein 1 A/1B-light chain 3 (LC3) is considered one of the most important regulators of autophagy [6]. LC3-I and LC3-II transformation acts as an important indicator for assessing autophagy activity [7]. mTOR, a serine/threonine kinase, is a master induction factor for autophagy [8,9]. mTOR was considered a major regulator of this signaling pathway, which regulates multiple biological processes, including cell proliferation, autophagy, and inflammation [10]. Deregulation of mTOR signaling was also involved in tumor formation and progression [11,12]. Autophagy can be regulated by many signaling pathways, including AKT/mTOR pathways, which are crucial in tumor initiation and progression. The accumulation of reactive oxygen species (ROS) plays an important role in apoptosis and autophagy [13,14], and ROS have been identified as one of the key factors for inducing tumor development [15]. Therefore, the mediating effect of ROS on the signaling pathway may be useful for cancer therapy.

Phytochemicals have become a promising approach in the management of malignancies [16,17]. Rhein is a natural anthraquinone found in several medicinal plants such as *Rheum undulatum, Rheum palmatum,* and *Cassia reticulata* [18] that exerts various pharmacological activities, including anti-inflammation, antioxidation, and anticancer activities [19,20]. Previous studies have confirmed that rhein is associated with autophagy in pancreatic cancer cells [21]. Previous studies have suggested that rhein suppressed non-small-cell lung cancer cell growth in vitro and in vivo by suppressing the STAT3 pathway [22] and induced HepaRG cell death through S-phase cell cycle arrest and apoptosis [23]. In addition, the anticancer activity of rhein has been reported in breast [24], ovarian [25], and colon cancers [26], suggesting that it could be a novel agent for OC prevention and treatment. However, the effects of rhein in OC cells and its underlying mechanisms have not been well studied.

In this study, we investigated the anticancer effects and underlying molecular mechanisms of rhein in YD-10B and Ca9-22 OC cells in vitro and in vivo. In this study, rhein significantly inhibited OC cell proliferation, migration, and invasion; -induced S-phase cell cycle arrest and apoptosis; -induced ROS generation; -and inhibited the autophagy of OC cells by inhibiting the mTOR signaling pathway.

## 2. Results

### 2.1. Rhein Exhibited Antiproliferative Effects in OC Cells

**Figure 1:**
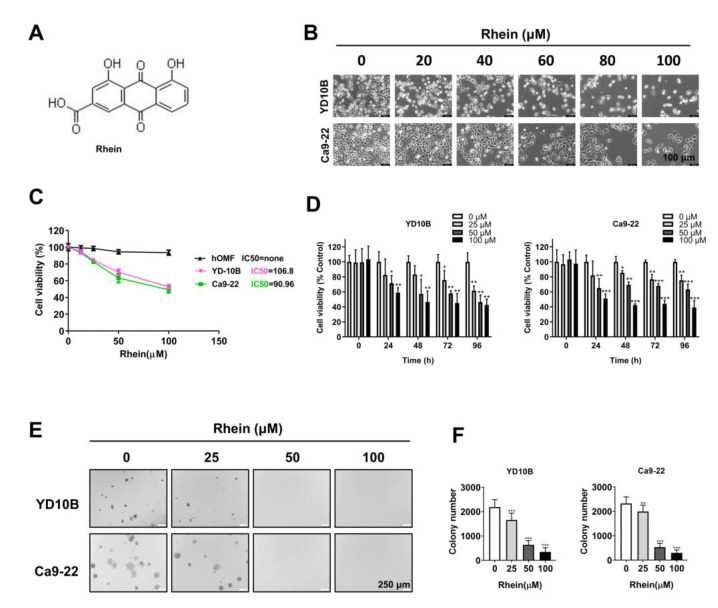
Rhein exhibits antiproliferative effects in OC cells. (**A**) Chemical structures of rhein. (**B**) The cell morphology of YD-10B and Ca9-22 cells after treatment with the indicated concentrations of rhein for 48 h was observed under a light microscope (magnification, 100×). (**C**) The IC50 value of oral cancer cells (YD-10B and Ca9-22) and normal oral epithelial cell (hOMF) was examined by CCK-8 assay at 24 h. (**D**) OC cells were treated with 0, 25, 50, and 100 μM rhein for 0, 24, 48, 72, and 96 h. Cell viability was measured with CCK-8 assay. (**E**) Effects of rhein on colony formation in YD-10B and Ca9-22 cells (magnification, 50×). (**F**) Quantitative graphs of colonies formation. * *p* < 0.05; ** *p* < 0.01; *** *p* < 0.001.

To evaluate the effect of rhein (structure as shown in Figure 1A) on the growth of human OC cells, YD-10B and Ca9-22 cells were treated with different concentrations (0, 20, 40, 60, 80, and 100 μM) of rhein for 48 h. As shown in Figure 1B, the cell density was significantly reduced by the rhein treatment. The majority of OC cells perished when exposed to 100 μM rhein. Thus, rhein concentrations of 0, 25, 50, and 100 μM were selected in the following experiments. Then, we detected the effect of different concentrations of rhein (0–100 μM) on OC cells YD-10B, Ca9-22, and normal oral epithelial cell hOMF by CCK-8 assay. As the results show, the IC50 of rhein in YD-10B and Ca9-22 are 106.8 and 90.96 μM respectively, and there is no significantl effect on the proliferation of hOMF cells (Figure 1C). Thus, the concentrations 0, 25, 50, and 100 μM rhein were selected in the following experiments. Cell proliferation was analyzed using the CCK-8 assay. As shown in Figure 1D, rhein inhibited OC cell proliferation with a dose-dependent manner. The soft agar colony formation assay showed that the number and size of clones in YD-10B and Ca9-22 cells were markedly reduced by rhein treatment in a dose-dependent manner (Figure 1D,E). These results indicate that rhein effectively inhibits the growth of OC cells.

### 2.2. Rhein Inhibits the Migration and Invasion of OC Cells

**Figure 2:**
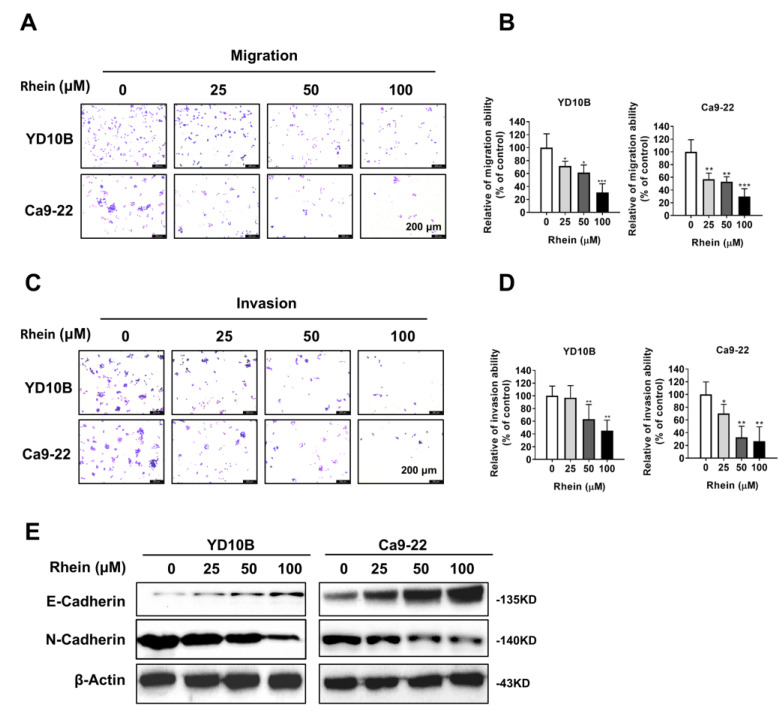
Rhein inhibits the migration and invasion of OC cells. (**A**,**B**) The migration abilities of YD-10B and Ca9-22 cells were determined 48 h after treatment with the indicated concentrations of rhein. (**C**,**D**) The invasion abilities of YD-10B and Ca9-22 cells were determined 48 h after treatment with the indicated concentrations of rhein. (**E**) Western blot analysis of the expressions of E-cadherin and N-cadherin in YD-10B and Ca9-22 cells 48 h after treatment with the indicated concentrations of rhein. *p < 0.05; ** *p* < 0.01; *** *p* < 0.001.

Metastasis was the major cause of cancer death and treatment failure. To explore whether rhein could inhibit OC cell metastasis, Transwell migration and invasion assays were performed. Consequently, the migration and invasion ability of OC cells was significantly inhibited by rhein treatment in a dose-dependent manner (Figure 2A–D). The accumulated evidence showed that the epithelial–mesenchymal transition (EMT) process was associated with cancer cell growth and metastasis [27,28]. Thus, we detected the expression of EMT marker proteins E-cadherin and N-cadherin in OC cells by Western blotting assay 48 h after treatment with rhein. The results showed that E-cadherin was upregulated, whereas N-cadherin was downregulated by rhein treatment in both YD-10B and Ca9-22 cells (Figure 2E). These results indicate that rhein inhibits the migration and invasion of OC cells by regulating the EMT process.

### 2.3. Rhein Induces the S-Phase Cell Cycle Arrest of OC Cells

**Figure 3:**
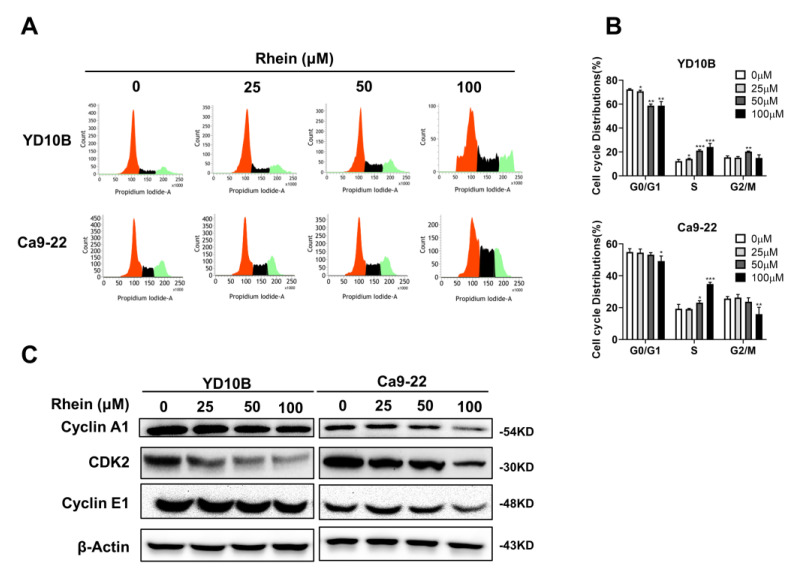
Rhein induces S-phase cell cycle arrest of OC cells. (**A**) The effects of rhein on cell cycle distribution were quantitated by flow cytometry. (**B**) Bar graph representing the quantified values of cells in different phases. (**C**) The protein expressions of cyclin A1, CDK2, and cyclin E1 were determined using Western blotting. * *p* < 0.05; ** *p* < 0.01; *** *p* < 0.001.

To explore the mechanism of rhein in the inhibition of the growth of OC cells, we evaluated the cell cycle distributions of YD-10B and Ca9-22 cells by flow cytometry following rhein (0, 25, 50, and 100 μM) treatment for 48 h. The results indicated that rhein significantly increased the proportion of S-phase cells in YD-10B and Ca9-22 cells (Figure 3A,B). Then, the effects of rhein were examined using the expression of S-phase-associated proteins, including cyclin A1, cyclin E1, and CDK2, by Western blot analysis. As shown in Figure 3C, rhein downregulated the expression levels of cyclin A1, cyclin E1, and CDK2 in YD-10B and Ca9-22 cells.

### 2.4. Rhein Induces the Apoptosis of OC Cells

**Figure 4:**
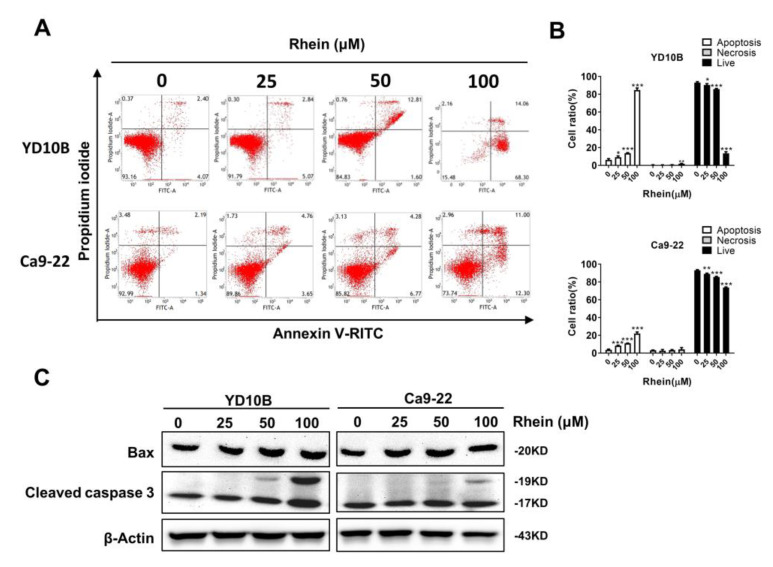
Rhein induces apoptosis of OC cells. (**A**,**B**) The effects of rhein on YD-10B and Ca9-22 cell apoptosis were quantitated by flow cytometry. (**C**) The effects of rhein on the expression levels of Bax and cleaved caspase-3 were determined by Western blotting. * *p* < 0.05; ** *p* < 0.01; *** *p* < 0.001.

To determine the effect of rhein on apoptosis of OC cells, YD-10B and Ca9-22 cells were treated with 0, 25, 50, and 100 μM rhein for 48 h, and the results showed that rhein dose-dependently induced OC cell apoptosis (Figure 4A,B). Rhein induced apoptosis was further evidenced by the increase in apoptotic markers Bax and cleaved caspase-3 (Figure 4C). Collectively, these results indicated that rhein can trigger apoptosis to suppress the proliferation of OC cells.

### 2.5. Rhein Induces ROS Generation and Suppresses the Akt/mTOR Signaling Pathway in OC Cells

**Figure 5:**
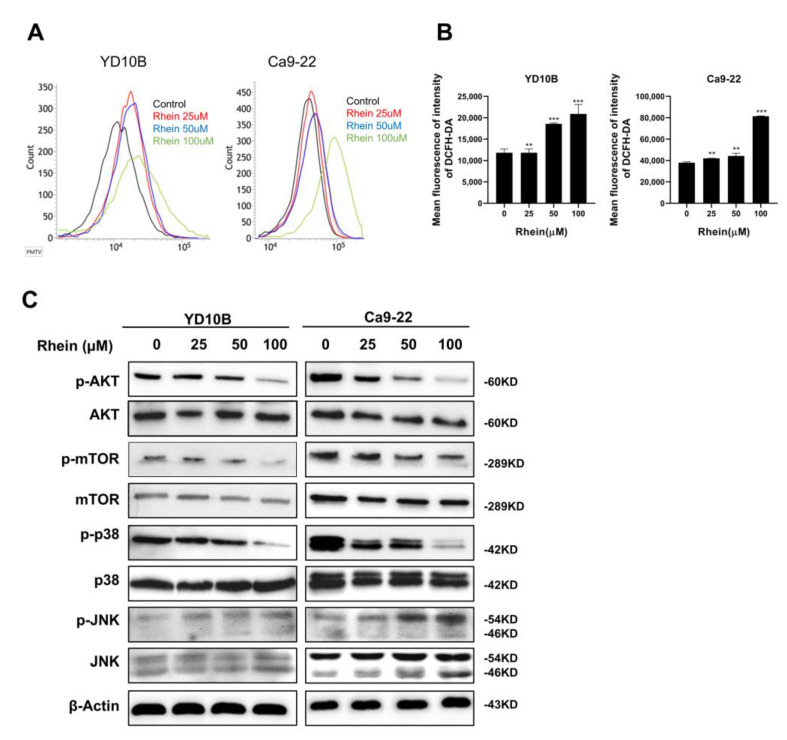
Rhein induces ROS generation and suppresses the AKT/ mTOR signaling pathway in OC cells. (**A**) Cells were treated with increased concentrations of rhein for 48 h, followed by incubating with 10 μM DCFH-DA for 30 min. The ROS level was determined by flow cytometry. (**B**) Quantitative analysis of the fluorescence intensity is shown in histograms. (**C**) The expression levels of AKT, p-AKT, mTOR, p-mTOR, p38, p-p38, JNK, and p-JNK were determined by Western blotting. ** *p* < 0.01; *** *p* < 0.001.

Increasing evidence has shown that ROS levels are involved in cellular apoptosis and cancer progression. To explore whether rhein can induce the ROS level of OC cells, YD-10B and Ca9-22 cells were treated with 0, 25, 50, and 100 μM rhein for 24 h. The production of ROS was measured using a 2′,7′-fichlorofluorescein diacetate probe (Merck KGaA, Darmstadt, Germany) and then analyzed through flow cytometry. As the results showed, the ROS level was increased by treating YD-10B and Ca9-22 cells with rhein (Figure 5A,B). The AKT/mTOR signaling pathway plays a crucial role in tumorigenesis [29,30]. Then, we determined whether rhein had any effect on the AKT/mTOR signaling pathway. As presented in Figure 5A, rhein treatment suppressed p-AKT, p-mTOR, and p-p38 expression levels and upregulated JNK expression levels in YD-10B and Ca9-22 cells. These results suggest that rhein inhibits OC cell growth by inducing ROS generation and inactivating the AKT/mTOR signaling pathway.

### 2.6. Rhein Inhibits OC Cell Growth in a Xenograft Mouse Model

**Figure 6:**
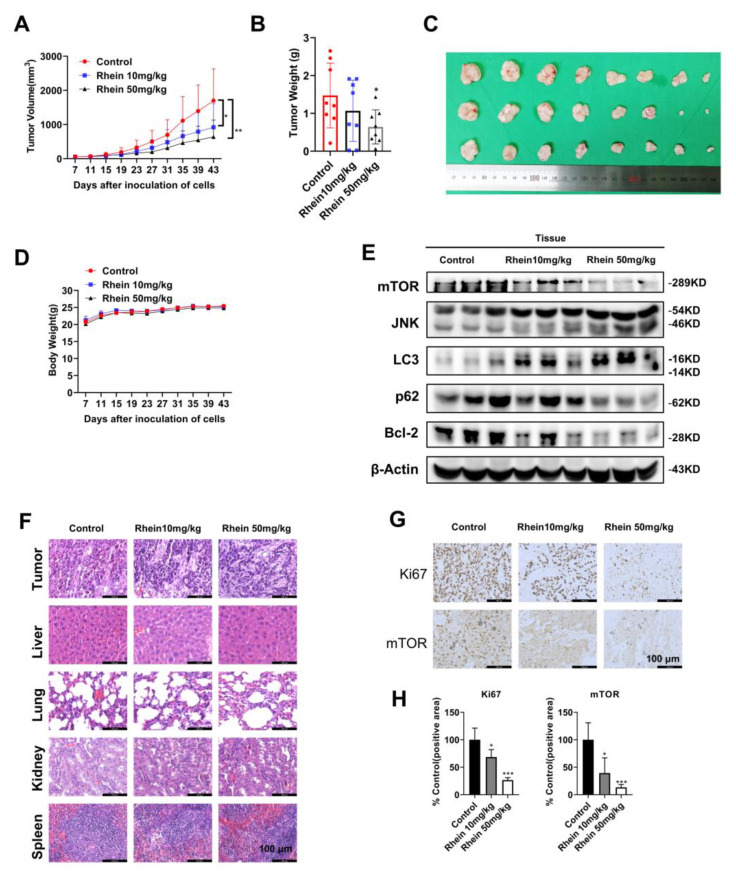
Rhein inhibits the growth of OC cells in a xenograft mouse model. (**A**) Ca9-22 cells were injected subcutaneously into the right flank of BALB/c-nu mice. After 7 days, mice were randomly allocated into 3 groups for the intraperitoneal administration of the vehicle, rhein 10 mg/kg, and rhein 50 mg/kg 3 times a week. The tumor volume was measured every 2 days, and the average tumor volumes in mice were measured. (**B**) Rhein significantly inhibited the growth of tumor xenografts. (**C**) Images of xenograft tumor volume. (**D**) Body weights were measured every 4 days. (**E**) The expression of mTOR, JNK, LC3, p62, and Bcl-2 was assessed by Western blotting. (**F**) H&E staining results showed no significant organ-related toxicities. (**G**,**H**) Immunohistochemical analysis of Ki67 and mTOR in xenograft tumor tissues. * *p* < 0.05; ** *p* < 0.01; *** *p* < 0.001.

**Figure 7:**
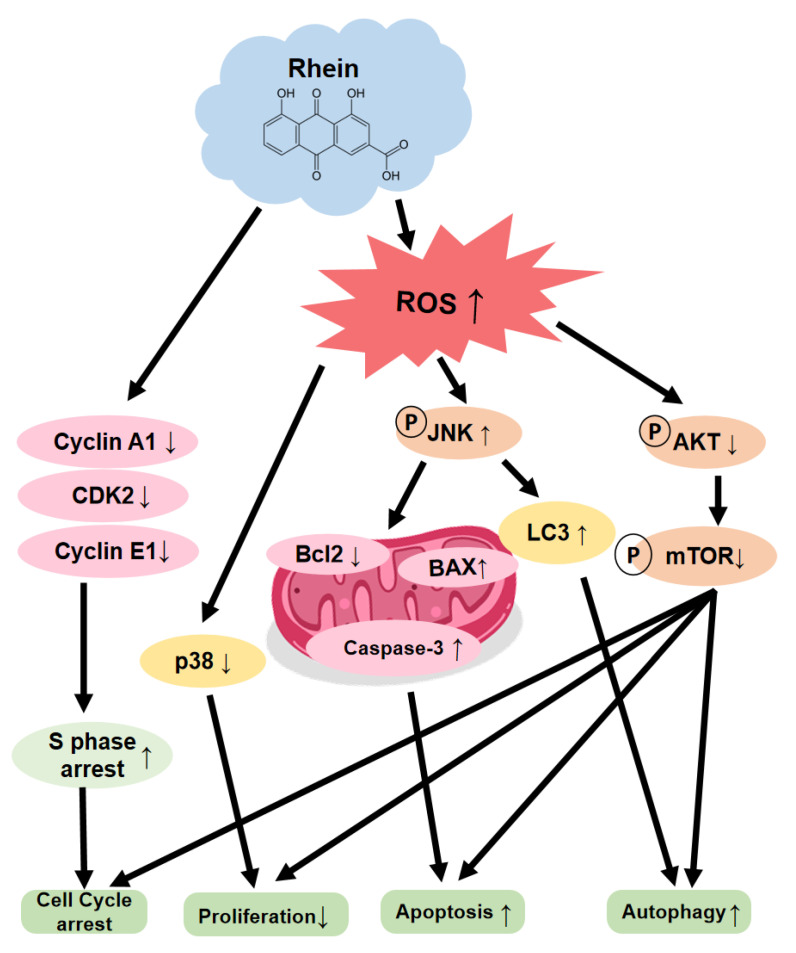
Rhein induces OC cell apoptosis and ROS via suppressed AKT/mTOR signaling pathway.

A tumor xenograft mouse model was used to assess the anticancer activity of rhein in vivo. Tumor-bearing mice received intraperitoneal injections of saline and two dosages of rhein (10 and 50 mg/kg) 3 times a week for 36 days. As observed, the rhein-treated group (10 or 50 mg/kg bodyweight) had significantly inhibited tumor growth compared with the vehicle-treated group (Figure 6A–C). Furthermore, the weight of mice was monitored every 4 days, and the rhein administration did not affect it (Figure 6D). The histological structure of the liver, lung, kidney, and heart was examined after 36 days of rhein treatment in tumor-bearing mice. As observed, the histological structure of the liver, lung, kidney, and heart had no significant changes compared with the vehicle group (Figure 7F). These results demonstrated that rhein could effectively inhibit tumor growth without exhibiting obvious toxicity. To further confirm whether the results of in vitro experiments were consistent with those in vivo, tumor tissues of tumor-bearing mice were collected for immunohistochemical (IHC) staining and Western blotting. According to the results, the expression levels of mTOR, p62, and Bcl2 were significantly downregulated, and those of JNKand LC3 were upregulated after treatment with rhein (Figure 6E). In addition, IHC results showed that protein levels of Ki67 and mTOR were downregulated after treatment with rhein, which was consistent with in vitro data (Figure 6F,H). Overall, these results demonstrated that rhein effectively inhibited OC tumor growth through the mTOR pathway in vivo and has the potential to be used as a chemotherapeutic agent for OC.

## 3. Discussion

OC still has a relatively high incidence and poor prognosis. Searching for chemotherapeutic adjuvant drugs that work through various anticancer mechanisms may lead to new targeted agents and therapeutic strategies. New treatment methods and effective drugs are necessary to conquer this disease. This study demonstrated that rhein significantly inhibits OC cell proliferation, colony formation, migration, and invasion while inducing apoptosis and S-phase cell cycle arrest. Our results reveal that rhein induces OC cell apoptosis and autophagy, which is regulated by ROS generation and the mTOR signaling pathway.

OC is one of the common head and neck tumors. Although there are perfect treatment measures, the prognosis is not satisfactory because of drug resistance, local invasion, and metastasis. EMT is a biological process in which epithelial cells transform into cells with mesenchymal characteristics and enhance the ability of cell migration. Studies have shown that EMT can promote the development and metastasis of tumor cells [31], and E-cadherin and N-cadherin are two marker proteins in EMT. E-cadherins are a type of cell adhesion molecule that can specifically bind to Ca9-22 to exert the cell adhesion function. Studies have found that N-cadherin can promote tumor cells to detach from the primary tumor site and adhere to endothelial or stromal components, transforming epithelial cells into mesenchymal cells, thereby acquiring an aggressive phenotype and being prone to distant metastasis and malignant evolution [32]. Several studies have shown that the low expression of E-cadherin and abnormal expression of N-cadherin are related to tumor invasion and metastasis [33,34]. Through the results of the transwell assay and Western blot, we found that rhein effectively inhibited the EMT process by upregulating E-cadherin and downregulating N-cadherin to control the migration and invasion of cancer cells (Figure 2A–E).

ROS is a key regulatory molecule of various cell signals, and ROS levels play a crucial role in regulating tumor cell apoptosis, autophagy, proliferation, and differentiation [35] [36]. Many studies have reported that natural plant compounds kill tumor cells by inducing intracellular ROS production [37,38]. As studies have shown, arsenic, the main active ingredient of realgar, induces autophagy and apoptosis in cancer cells by generating ROS [39]. In this study, we found that rhein can induce ROS production in OC cells and then increase the apoptosis marker Bax and cleaved caspase-3 to trigger apoptosis, which further proves that rhein can trigger apoptosis to inhibit the proliferation of OC cells (Figure 4A–C). In addition, ROS can regulate various signaling pathways, such as p38 and JNK members of the MAPK family, which can regulate various cell death mechanisms, such as autophagy and apoptosis [40,41]. Our study showed that rhein activated the JNK pathway and inhibited the AKT/mTOR pathway by inducing ROS accumulation in OC.

Autophagy is a physiological process in which lysosomes degrade intracellular proteins and organelles in eukaryotic cells. Many diseases, including cancer, are closely related to autophagy [42]. LC3 and p62 are autophagy-related signature proteins. When autophagy is activated, LC3 will be converted into cytoplasmic LC3 (LC3I), modified into membrane-bound LC3 (LC3II), bound to the autophagosome membrane simultaneously, and the adapter protein p62 of selective autophagy can connect LC3II and ubiquitinated substrates and be degraded in autolysosomes [43]. The results of this study showed that rhein treatment induced a significant downregulation of p62 expression and an upregulation of LC3 expression (Figure 6E). Studies have shown that the Akt/mTOR signaling pathway is also a negative regulatory pathway for autophagy, and when the pathway is activated in a phosphorylated form, autophagy is inhibited [44]. The results of this study show that rhein can inhibit the expression of p-Akt/Akt and p-mTOR/mTOR-related proteins in the Akt/mTOR signaling pathway in OC cells and mouse tumor tissues and induce autophagy, suggesting that rhein can induce autophagy by inducing the mechanism by which autophagy plays an anticoagulant role, which is related to the inhibition of the Akt/mTOR signaling pathway by rhein (Figure 7).

Natural products such as paclitaxel, camptothecin, and vinblastine play important roles in the clinical treatment of cancers [45]. Many studies have demonstrated that rhein has a powerful therapeutic effect by inhibiting cancer-related signaling pathways in numerous cancer cells [46,47,48]. However, only one study has reported on the anticancer effects of rhein on OC cells, which indicated that rhein inhibited OC cell growth through a decrease in MMP-2 and VEGF protein levels [49]. In this study, we explored the anticancer activity of rhein in OC cells through various in vitro experiments. The activation of the apoptosis pathway is one of the main strategies for cancer treatment. Our results revealed that rhein increased cleaved caspase-3 and cleaved PARP expression in OC cells, which indicates that rhein induced the apoptosis of OC cells. Meanwhile, cell cycle deregulation is one of the characteristics of cancer cells; thus, blocking the cell cycle of cancer cells is also one of the strategies for cancer treatment. CDK/cyclins have crucial roles in the regulation of cell cycle progression and other major biological processes [50]. Cyclin A1, cyclin E1, and CDK2 are regulators of the S-phase [51]. In the present study, we found that rhein inhibits OC cell growth by inducing S-phase cell cycle arrest and that the molecular mechanisms were related to the downregulation of cyclin A1, cyclin E1, and CDK2. These results indicate that rhein is a potential therapeutic agent for OC treatment through the activation of apoptosis and blocking of the cell cycle of OC cells.

## 4. Materials and Methods

### 4.1. Reagents and Antibodies

Rhein (purity: >98%) was obtained from Harvey Biotech Co., Ltd. (Beijing, China). The different working concentrations of rhein were dissolved in dimethyl sulfoxide (DMSO). The primary antibodies E-cadherin (67A4), N-cadherin (13A9), and β-actin (C4) were obtained from Santa Cruz Biotechnology (Santa Cruz Biotechnology, Inc., Dallas, TX, USA). Cyclin D1 (#55506), CDK2 (#18048), cyclin E1 (#4129), cleaved caspase-3 (#9661), Bax (#2772), phospho-AKT (#9271), AKT (#9272), phospho-JNK (#G9), JNK (#9252), phospho-p38 (#28B10), p38 (#9212), Phospho-mTOR (#5536), mTOR (#2983), LC3A/B (#D9U4C), p62 (#88588), and Bcl-2 (#15071) were purchased from Cell Signaling Technology (Cell Signaling Technology, Danvers, MA, USA). 3-Methyladenine (3-MA) was purchased from Sigma–Aldrich Merck, Darmstadt, Germany.

### 4.2. Cell Culture

Ca9-22 cells were purchased from the Japanese Collection of Research Bioresources Cell Bank (Shinjuku, Japan). YD-10B cells were obtained from the Oral Cancer Institute at the College of Dentistry, Yonsei University (Seoul, Republic of Korea) [52]. The cells were incubated in Dulbecco’s modified Eagle’s medium (Gibco™, Grand Island, NY, USA) with 10% fetal bovine serum (GenDEPOT, Katy, TX, USA) and 1% penicillin/streptomycin (Gibco). Cells were maintained at 37 °C in a 5% CO_2_ incubator.

### 4.3. Cell Viability Assay

CCK-8 assays were performed to assess cell viability. Briefly, OC cells were plated on 96-well plates at a density of 1000 cells/well. After 24 h of culturing, the medium was replaced with a new medium containing specified concentrations of rhein. Then, the cells were cultured for 0, 24, 48, 72, and 96 h and then incubated with 10 µL of the CCK-8 regent per well for another 1 h at 37 °C. The optical density of each well at 450 nm was measured using a microplate reader (BioTek Instruments, Winooski, VT, USA).

### 4.4. Soft Agar Colony Formation Assay

YD-10B and Ca9-22 cells (8 × 10^3^ cells/well) were suspended in a complete growth medium containing 0.3% agar with specified concentrations of rhein and then overlaid into 6-well plates containing 0.6% agar and specified concentrations of rhein. The cultures were incubated for 14 days in a 5% CO_2_ incubator at 37 °C. Then, photographs were taken under a microscope (Leica), and the number of colonies was counted using ImageJ.

### 4.5. Cell Cycle and Apoptosis Analysis

OC cells were seeded into 60-mm culture dishes (1 × 10^5^ cells/dish) and cultured overnight at 37 °C. Then, the cells were treated with the indicated concentrations of rhein for 48 h. To analyze the cell cycle, cells were collected after they were digested by trypsin and centrifuged at 1000 rpm for 5 min. Then, the cells were washed with cold phosphate-buffered saline (PBS) twice and fixed in 70% ethanol at −20 °C overnight. Cells were centrifuged at 2000 rpm for 5 min, washed with PBS, and incubated with propidium iodide (PI, 20 μg/mL) and RNase (100 μg/mL) in the dark for 30 min. Thereafter, the cell characteristics were detected and measured by flow cytometry. To analyze apoptosis, the cells were stained with Annexin V-FITC (BioLegend, San Diego, CA, USA) and PI (20 μg/mL) in the dark for 20 min and subsequently analyzed using FACS Verse flow cytometry (BD Science, San Jose, CA, USA).

### 4.6. Invasion and Migration Assays

Migration assays were performed in 24-well Transwell plates (8 µm pore size, Corning) according to the manufacturer’s instructions. Cells were seeded in the upper chambers at a density of 8 × 10^4^ cells in 100 μL of serum-free DMEM with the indicated concentrations of rhein. The lower chambers were filled with 600 μL of the culture medium containing 10% FBS to stimulate cell movement. After 48 h of culturing, the cells were fixed using 4% paraformaldehyde for 20 min, the non-invaded cells were removed using a cotton swab, whereas the invaded cells were stained with 0.05% crystal violet. For invasion assays, the chamber was pre-coated with Matrigel^®^, before repeating the same steps as for the migration assay. The invaded cell numbers were quantified by counting the stained cells under a microscope.

### 4.7. Measurement of Intracellular ROS 

2′,7′-Dichlorodihydrofluorescein diacetate (DCFH-DA) was used to detect intracellular ROS levels. Cells were seeded into 60 -mm culture dishes (1 × 10^5^ cells/dish). After incubating for 12 h, cells were treated with different concentrations of rhein (0, 25, 50, and 100 μM) for 24 h. Cells were washed with PBS and incubated in a fresh medium containing DCFH-DA (10  µg/mL) at 37 °C for 30 min. Then, the cells were collected and analyzed at excitation and emission wavelengths of 488 and 530 nm, respectively, by a flow cytometer.

### 4.8. Western Blotting Assay

OC cells (1 × 10^6^) were plated in 10 -cm dishes and incubated with 0, 25, 50, and 100 µM rhein at 37 °C for 48 h. Subsequently, the cells were collected and lysed using the PRO-PREP™ lysis buffer (Intron Biotechnology, Seongnam, Republic of Korea). The protein concentrations were measured by NanoDrop™ 2000 (Thermo Fisher Scientific, Waltham, MA, USA). A total of 30 µg protein was loaded, separated through sodium dodecyl-sulfate polyacrylamide gel electrophoresis, and then transferred to a polyvinylidene difluoride (PVDF) membrane (0.22  µm, Merck Millipore). The membranes were blocked in 5% BSA for 1 h and then incubated with the corresponding primary antibodies at 4 °C overnight, followed by horseradish peroxidase-conjugated secondary antibodies for 1 h at room temperature. The protein signals were detected with an ECL detection kit (GE Healthcare, Seoul, Republic of Korea) using a Da Vinci Fluorescence Imaging System (Da Vinci-K, Seoul, Republic of Korea). β-Actin was used as the loading control.

### 4.9. Xenograft Tumor Model in Mice

All animal experiments were performed according to the guidelines and approval of Kyungpook National University. Male athymic nude mice (4–5 weeks) were purchased from Charles River Technology. To establish CRC xenografts, Ca9-22 cells (5 × 10^6^ cells) suspended in 200 μL of PBS were subcutaneously injected into the flank of the mice. After 7 days of implantation, mice were randomly divided into 3 groups consisting of eight mice per group. Two groups were treated with rhein at 10 mg/kg and 50 mg/kg bodyweight (dissolved in 5% DMSO and 10% Tween-20 in PBS), and the third group was treated with vehicle only. Rhein or the vehicle was intraperitoneally injected three times a week for 36 days. The tumor volume and bodyweights were measured every 4 days. The tumor volume was calculated using the following ellipsoid formula: tumor volume (mm^3^) (length × width × height × 0.52). 

### 4.10. IHC Staining

To inhibit the endogenous peroxidase activity, tissue slices were submerged in 10% hydrogen peroxide for 15 min. Then, antigens were retrieved by heat treatment in citrate buffer (pH 6.0). Primary antibodies were incubated overnight at 4 °C (Ki-67, 1:200, mTOR, 1:200) and then incubated with a biotin-conjugated secondary antibody for 1 h at 37 °C. Images were visualized under a microscope and analyzed using ImageJ (v. 4). 

### 4.11. Statistical Analysis

The results are presented as the mean ± SD from three independent experiments. Statistical significance was determined using Student’s *t*-test. A *p*-value of < 0.05 was considered statistically significant.

## 5. Conclusions

Rhein significantly inhibited OC cell growth by inducing apoptosis and S-phase cell cycle arrest. Rhein inhibited OC cell migration and invasion through the regulation of EMT-related proteins. Rhein induced the ROS of OC cells to inhibit the AKT/mTOR signaling pathway. Taken together, we confirmed the anticancer activity of rhein in OC cells through the mTOR signaling pathway. These findings highlight the potential of rhein as a therapeutic agent for OC treatment.

## Data Availability

All relevant data were contained within the manuscript.
